# The First Homologous Expression System for the β-Lytic Protease of *Lysobacter capsici* VKM B-2533^T^, a Promising Antimicrobial Agent

**DOI:** 10.3390/ijms23105722

**Published:** 2022-05-20

**Authors:** Irina Kudryakova, Alexey Afoshin, Elena Leontyevskaya, Natalia Leontyevskaya (Vasilyeva)

**Affiliations:** Laboratory of Microbial Cell Surface Biochemistry, G. K. Skryabin Institute of Biochemistry and Physiology of Microorganisms, Russian Academy of Sciences, Pushchino Center for Biological Research, 5 Prosp. Nauki, 142290 Pushchino, Russia; kudryakovairina@yandex.ru (I.K.); alex080686@mail.ru (A.A.); ealeont@gmail.com (E.L.)

**Keywords:** homologous expression system, *Lysobacter*, staphylolytic β-lytic protease, GroEL(A) and T5 promoters, bacteriolytic enzymes

## Abstract

A successful homologous expression system based on *Lysobacter capsici* VKM B-2533^T^ and the plasmid pBBR1-MCS5 was first developed for a promising bacteriolytic enzyme of this bacterium, β-lytic protease (Blp). In the expression strains, *blp* gene expression under the regulation of the GroEL(A) and T5 promoters increased by 247- and 667-fold, respectively, as compared with the wild-type strain. After the cultivation of the expression strains *L. capsici* P_GroEL(A)_-*blp* and *L. capsici* P_T5_-*blp*, the Blp yield increased by 6.7- and 8.5-fold, respectively, with respect to the wild-type strain. The cultivation of the expression strain *L. capsici* P_T5_-*blp* was successfully scaled up. Under fermentation conditions the yield of the enzyme increased by 1.6-fold. The developed homologous system was used to express the gene of the bacteriolytic serine protease (Serp) of *L. capsici* VKM B-2533^T^. The expression of the *serp* gene in *L. capsici* P_T5_-*serp* increased by 585-fold. The developed homologous system for the gene expression of bacteriolytic *Lysobacter* enzymes is potentially biotechnologically valuable, and is promising for creating highly efficient expression strains.

## 1. Introduction

The β-lytic protease (Blp) is an extracellular bacteriolytic enzyme that is produced by some members of the genus *Lysobacter*. This enzyme was described in 1965, together with the α-lytic protease [1]. Both enzymes were isolated from the Gram-negative bacterium *Myxobacter* 495, now called *L. enzymogenes* ATCC 29487 [2]. These enzymes were discovered 40 years after the discovery of lysozyme, the first bacteriolytic enzyme. Bacteriolytic enzymes are produced by all living organisms, but the most active producers are bacteria. All bacteriolytic enzymes destroy peptidoglycan—the main structural component of bacterial cell walls. However, they differ in their specificity of action against peptidoglycan [3]. Enzymes that break the bond between *N*-acetylglucosamine and *N*-acetylmuramic acid in peptidoglycan are called muramidases (such as lysozyme). Glucosaminidases destroy the same bond as muramidases, to release *N*-acetylglucosamine at the reducing end. Enzymes that break the bond between the first amino acid of the peptide stem and the *N*-acetylmuramic acid of peptidoglycan are called amidases. Enzymes that destroy peptide bonds in the peptide subunits of peptidoglycan are bacteriolytic proteases. Thus, bacteriolytic enzymes are specific to all peptidoglycan bonds. This property makes bacteriolytic enzymes promising antimicrobial agents.

To date, however, only lysozyme has been widely used in medicine, agriculture, and the food industry. At the same time, many bacteriolytic enzymes, Blp included, are not inferior to, and even surpass the lytic action of lysozyme, but are not used widely in practice [4,5,6]. This is primarily due to the difficulties of obtaining effective expression systems for such enzymes, which include the “toxicity” of target proteins for producer cells, the absence of secretory pathways in recombinant strains that may be necessary for the correct folding of target proteins, and refolding problems in obtaining proteins from inclusion bodies [7,8,9,10]. A successful solution to this problem, in our opinion, could be the use of native producers of bacteriolytic enzymes to create biotechnologically significant homologous expression systems.

For the past 50 years, bacteriolytic enzymes of some representatives of the genus *Lysobacter* have been studied in our laboratory. Our main works investigate such enzymes in *Lysobacter* sp. XL1 [11,12,13,14,15,16,17]. Our recent research has been related to the study of antimicrobial potential in *L. capsici* VKM B-2533^T^ [6,10]. Thus, it has been found that this bacterium has a potent antimicrobial effect against bacteria, fungi, and yeasts. A number of enzymes possessing bacteriolytic activities have been isolated from the culture liquid of *L. capsici.* They include Blp, which has shown to have a potent lytic effect against living cells of clinical isolates of *Staphylococcus aureus* MRSA (the MIC of the enzyme being 2.85 µg/mL). The practical value of the enzyme is obvious, and we set a goal to develop a homologous expression system for Blp of *L. capsici* VKM B-2533^T^. This is also relevant, considering the absence of practically significant expression systems for bacteriolytic enzymes of Gram-negative bacteria. 

## 2. Results

### 2.1. Search for an Efficient Promoter among Known and Studied Promotors to Develop a Homologous Expression System Based on L. capsici VKM B-2533^T^

The successful creation of an expression system requires an efficient promoter. To date, the genetics of *Lysobacter* are practically unstudied, which significantly complicates the genetic manipulations for this bacterium. Still, two *L. enzymogenes* promoters are already known and have been successfully applied in practice to obtain recombinant strains. These are HSAF [18] and GroEL, as well as GroEL that has been modified by increasing the distance from the start codon to the Shine–Dalgarno region by the addition of nucleotide A, GroEL(A) [19]. To develop an expression system in *L. capsici*, we tested the GroEL and GroEL(A) promoters, as well as the promoter of bacteriophage T5, which was used for the first time in *Lysobacter* cells. The green fluorescent protein (GFP) was used as a marker to determine the efficiency of the promoters in *Lysobacter*.

**Figure 1:**
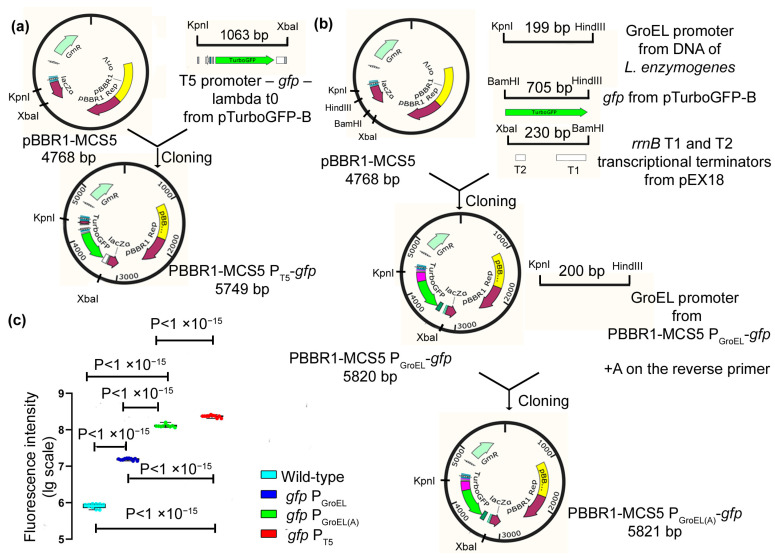
Construction of expression vectors PBBR1-MCS5 P_GroEL_-*gfp*, PBBR1-MCS5 P_GroEL(A)_-*gfp*, and PBBR1-MCS5 P_T5_-*gfp*. (**a**) Scheme for constructing expression vector PBBR1-MCS5 P_T5_-*gfp*. (**b**) Scheme for constructing expression vectors PBBR1-MCS5 P_GroEL_-*gfp* and PBBR1-MCS5 P_GroEL(A)_-*gfp*. (**c**) Measurement of fluorescence intensity in cells of *L. capsici* strains. The boxplots show the median and interquartile range (IQR). The values were obtained in three independent experiments: two independent experiments with two technical replicates and one independent experiment with six technical replicates. Statistical analysis was performed using a one-way ANOVA with Tukey’s multiple comparison test, *p* < 1 × 10^−15^, *F*(3, 36) = 6712.

As a basis for the construction of expression vectors, the pBBR1-MCS5 plasmid was used, and it is currently the only known plasmid that is maintained in *Lysobacter* cells. As a result, the expression vectors PBBR1-MCS5 P_GroEL_-*gfp*, PBBR1-MCS5 P_GroEL(A)_-*gfp*, and PBBR1-MCS5 P_T5_-*gfp* were obtained, which contained the GFP protein gene under the regulation of the chosen promoters (Figure 1a,b). The obtained vectors were transferred into *L. capsici* cells using electroporation (Methods). As a result, recombinant strains of *L. capsici* P_GroEL_-*gfp*, *L. capsici* P_GroEL(A)_-*gfp* and *L. capsici* P_T5_-*gfp* were obtained. 

To test the efficiency of the promoters, we measured the intensity of GFP fluorescence in cells of the expression strains (Figure 1c, Supplementary Table S1). Cells of all recombinant strains were shown to possess fluorescence, which was indicative of the work of all of the studied promoters. The highest fluorescence level was registered in cells of the strain *L. capsici* P_T5_-*gfp*, in which GFP is under the regulation of the T5 promoter (Figure 1c). The fluorescence intensities in the cells of this strain were 30 and 13 times higher than that in *L. capsici* P_GroEL_-*gfp* and *L. capsici* P_GroEL(A)_-*gfp*, respectively. This result indicates that the highest level of GFP expression occurs under the regulation of the T5 promoter. 

To develop a homologous expression system for Blp, we chose the expression vectors PBBR1-MCS5 P_GroEL(A)_-*gfp* and PBBR1-MCS5 P_T5_-*gfp* containing the GroEL(A) and T5 promoters, respectively. 

### 2.2. Development of a Homologous Expression System for Blp

Based on the chosen vectors, we constructed the vectors PBBR1-MCS5 P_GroEL(A)_-*blp* and PBBR1-MCS5 P_T5_-*blp*, in which the gene of the protein is under the regulation of the GroEL(A) and T5 promoters, respectively. These vectors were transferred into *L. capsici* VKM B-2533^T^ cells, and two expression strains *L. capsici* P_GroEL(A)_-*blp* and *L. capsici* P_T5_-*blp* were thus obtained (Methods).

The expression strains were cultivated on LB-M medium for 20 h; in the culture liquid, the bacteriolytic activity with respect to *S. aureus* 209 P living cells was measured (Table 1).

**Figure 2:**
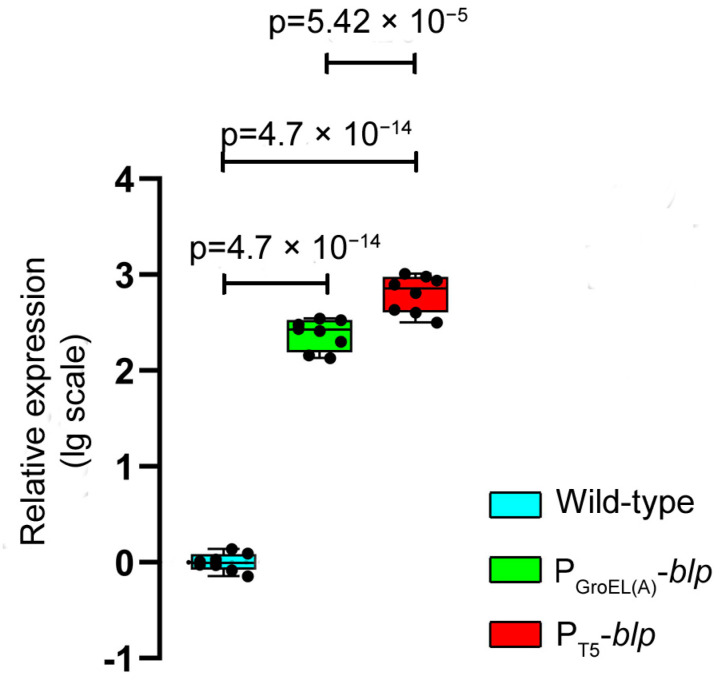
Relative level of expression of the *blp* gene in *L. capsici* strains. The boxplots show the median and IQR. The values were obtained in four independent experiments, each with two technical replicates. Statistical analysis was performed using a one-way ANOVA with Tukey’s multiple comparison test, *p* < 1 × 10^−15^, *F*(2, 21) = 769.

As seen from the table, the bacteriolytic activity in the culture liquid of the expression strains *L. capsici* P_GroEL(A)_-*blp* and *L. capsici* P_T5_-*blp* increased by 3.02- and 3.86-fold, respectively, as compared with the wild-type strain. A comparative analysis using the RT-qPCR method showed that the level of expression of the *blp* gene in *L. capsici* P_GroEL(A)_-*blp* and *L. capsici* P_T5_-*blp* was 246 and 667 times higher, respectively, than in *L. capsici* VKM B-2533^T^. Herewith, the level of expression of the *blp* gene under the regulation of the T5 promoter was 2.71 times as high as under the regulation of GroEL(A) promoter (Figure 2, Supplementary Table S2).

**Figure 3:**
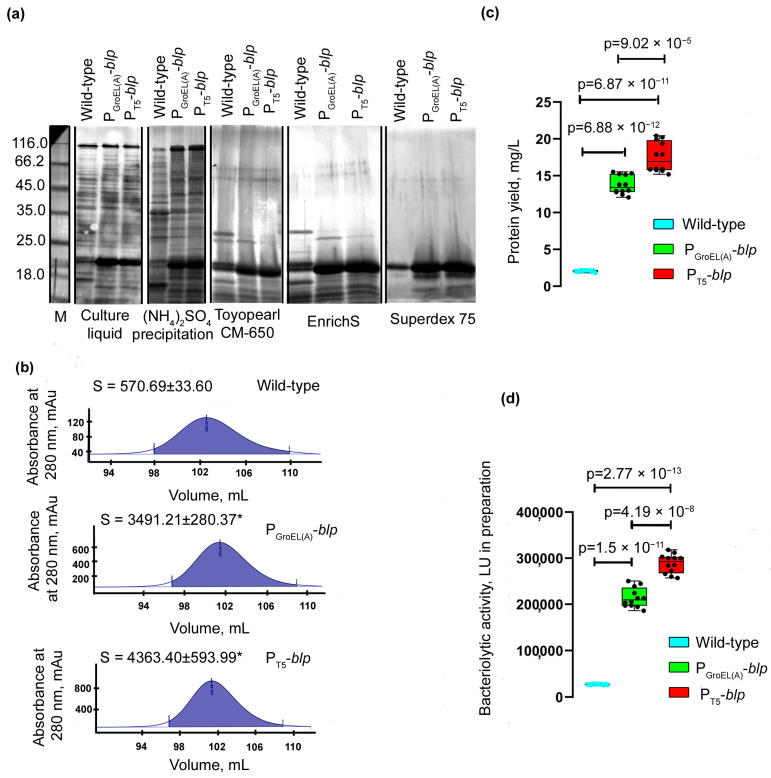
Assessment of efficiency of the developed homologous expression system. (**a**) SDS-PAGE: lanes correspond to samples of the culture liquid of *L. capsici* strains (Supplementary Figure S2); M and lanes correspond to samples after ammonium sulfate precipitation (Supplementary Figure S3); lanes correspond to samples after Toyopearl CM-650 (Supplementary Figure S3); lanes correspond to samples after EnrichS (Supplementary Figure S4); lanes correspond to samples after Superdex 75 (Supplementary Figure S5). The gel was stained with imidasole–ZnCl_2_ solutions. (**b**) β-lytic protease (Blp) peak area the purification on a Hiload 16/60 column (Superdex 75). The mean values were obtained in two independent experiments, each with two technical replicates. The two groups were compared using a two-sided Mann–Whitney *U*-test (*p* = 0.0286). (**c**) Comparison of the Blp yields in *L. capsici* strains. The boxplots show the median and IQR. The values were obtained in two independent experiments, each with six technical replicates. Statistical analysis was performed using a Welch ANOVA followed by Tamhane’s T2 test for multiple comparisons, *p* < 1 × 10^−15^, *W*(2, 14.82) = 827.8. (**d**) Comparison of the bacteriolytic activity in the culture liquid of *L. capsici* strains. The boxplots show the median and IQR. The values were obtained in two independent experiments, each with six technical replicates. Statistical analysis was performed using a Welch ANOVA followed by Tamhane’s T2 test for multiple comparisons, * *p* < 1 × 10^−15^, *W*(2, 14.72) = 1372.

To assess the yield of the Blp protein, it was purified from the culture liquid of *L. capsici* strains according to the previously developed scheme (Methods and Supplementary Figure S1). As seen from the electrophoregram (Figure 3a), all preparations of the expression strains obtained at different stages of purification had a higher content of protein in the region corresponding to the electrophoretic mobility of the Blp protein, as compared with similar preparations obtained during purification from the culture liquid of the wild-type strain. 

Comparison of the peak areas of the homogeneous Blp protein obtained at the final stage of purification showed them to be 6.1 and 7.7 times larger, respectively, in *L. capsici* P_GroEL(A)_-*blp* and *L. capsici* P_T5_-*blp* compared with the peak area of the Blp from wild-type *L. capsici* (Figure 3b). The yield of the Blp in *L. capsici* P_GroEL(A)_-*blp* was 13.755 mg/L, and in *L. capsici* P_T5_-*blp*, 17.519 mg/L, which was 6.7- and 8.5-fold greater, respectively, compared with wild-type *L. capsici* (Figure 3c, Table 2). The bacteriolytic activity of the Blp preparations obtained from *L. capsici* P_GroEL(A)_-*blp* and *L. capsici* P_T5_-*blp* strains was 7.8 and 10.5 times higher, respectively, compared with the Blp preparation obtained from the wild-type strain (Figure 3d, Table 2). In all of the preparations obtained, the specific activity of the Blp was an average of 38,902 LU/mg, which was indicative of their high degree of purity. 

Thus, the developed homologous expression systems for Blp under the regulation of the GroEL(A) and T5 promoters can be considered as being successful. 

### 2.3. A Feature of the Developed Homologous Expression Systems

**Figure 4:**
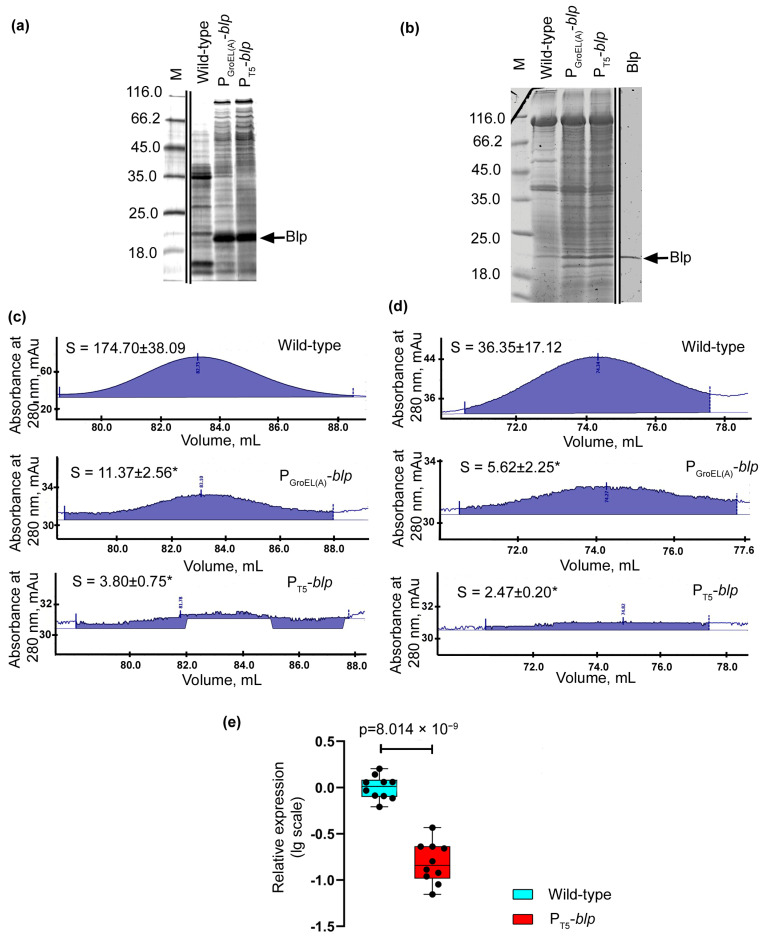
Comparative analysis of the production of some proteins in cells of *L. capsici* strains. (**a**) SDS-PAGE: Lanes correspond to samples of the culture liquid of *L. capsici* strains (Supplementary Figure S6). The gel was stained with imidasole–ZnCl_2_ solution. (**b**) SDS-PAGE: Lanes correspond to samples of *L. capsici* strain cells and purified Blp of *L. capsici* VKM B-2533^T^ (Supplementary Figures S7 and S8). The gel was stained with a solution of Coomassie Brilliant Blue R-250. (**c**) Peak area of protein L1 at purification on a Hiload 16/60 column (Superdex 75). The mean values were obtained in two independent experiments, each with two technical replicates. The two groups were compared using a two-sided Mann–Whitney *U*-test (*p* = 0.0286). (**d**) Peak area of a mixture of serine proteases at the purification on a Hiload 16/60 column (Superdex 75). The mean values were obtained in two independent experiments, each with two technical replicates. The two groups were compared using a two-sided Mann–Whitney *U*-test (*p* = 0.0286). (**e**) Relative level of expression of the *alpA* gene in *L. capsici* P_T5_-*blp*. The boxplots show the median and IQR. The values were obtained in four independent experiments, each with two technical replicates. The two groups were compared using an unpaired two-tailed Student’s *t*-test, *t* = 10.07, *df* = 18, * *p* = 0.0286.

As a result of the characterization of the developed homologous expression systems for Blp, it was noted that the profiles of the major proteins changed in the culture liquid preparations of the expression strains (Figure 4a). In the expression strains, only one major protein, Blp, was observed. We suggest that this was due to the secretory apparatus of the cell, which cannot cope with the increased load upon it. This is evidenced by some accumulation of a protein within the cells of the expression strains, which in terms of electrophoretic mobility, coincided with Blp (Figure 4b). The change in the profiles of the major proteins could also be associated with a change in the level of expression of secreted protein genes, resulting from genetic manipulation.

The proof of a decrease in the total production of secreted protein in cells of the expression strains was taken from the analysis of the areas of two protein peaks, which, as shown previously, corresponded to a mixture of serine proteases and bacteriolytic protein L1, and emerged before the peak of the Blp at the final stage of purification [10]. 

As a result, it was found that the peak area of the L1 protein in the strains *L. capsici* P_GroEL(A)_-*blp* and *L. capsici* P_T5_-*blp* was 15.4- and 46-fold less, respectively, compared to the similar peak obtained as a result of Blp purification from the wild-type strain (Figure 4c). The peak area of a mixture of serine proteases in the strains *L. capsici* P_GroEL(A)_-*blp* and *L. capsici* P_T5_-*blp* was 6.5- and 14.7-fold less, respectively, compared to the similar peak obtained as a result of purification of Blp from the wild-type strain (Figure 4d).

Using the RT-qPCR method, we revealed that the expression level of the *alpA* gene encoding the bacteriolytic enzyme L1 in strain *L. capsici* P_T5_-*blp* was 6.1-fold lower than in the wild-type strain (Figure 4e, Supplementary Table S3). Thus, the production of this protein in the cells of the recombinant strains decreased due to a decrease in the level of gene expression. 

It should be noted here that a decrease in the level of expression and secretion of other (native) bacteriolytic enzymes in cells of expressing *L. capsici* strains greatly facilitated the preparation of pure Blp. Obviously, this property increases the value of the developed homologous expression systems.

Thus, new expression systems for the promising bacteriolytic enzyme Blp, based on the *L. capsici* strain VKM B-2533^T^ and the expression plasmids PBBR1-MCS5 P_GroEL(A)_-*blp* and PBBR1-MCS5 P_T5_-*blp* were successfully developed. For the expression strain *L. capsici* P_T5_-*blp*, in which Blp is under the regulation of the T5 promoter, it was reliably established that the gene expression of this protein and its yield were higher as compared to the strain *L. capsici* P_GroEL(A)_-*blp*, in which Blp is under the regulation of the GroEL(A) promoter. 

### 2.4. Upscaling L. capsici P_T5_-blp Cultivation

It is well known that many expression systems for biotechnologically valuable proteins that have demonstrated good results under laboratory conditions have not been widely used because their cultivation was proven to be non-scalable. Blp is a potent staphylolytic enzyme, which makes it promising for its use in medicine. For this reason, we set ourselves the task of scaling-up the cultivation of an expression strain. We chose the strain *L. capsici* P_T5_-*blp* to solve this problem, because it has reliably shown the best parameters of Blp production.

Previously, our laboratory developed an RM medium that is optimal for the cultivation of *Lysobacter* spp. under the conditions of commercial fermentation [13]. First, we studied the dynamics of growth and the development of bacteriolytic activity during the cultivation of *L. capsici* P_T5_-*blp* on an RM medium under laboratory conditions (Supplementary Figure S9a). The maximum bacteriolytic activity (1997 LU/mL) was observed from 30 h of cultivation. By this time, the culture growth value was 3.21 at OD_540_. The culture growth and Blp production were stable. Thus, the RM medium was suitable for the fermentation of *L. capsici* P_T5_-*blp*.

To upscale the cultivation process, a 10 L ANKUM-2M fermenter (Special Design Bureau of the Russian Academy of Sciences, Pushchino, Russia) was used. A 50 mL culture of *L. capsici* P_T5_-*blp* grown in flasks of RM medium for 20 h was inoculated into 5 L of the same medium containing 20 μg/mL of the antibiotic Gm. Fermentation was conducted at a temperature of 29 °C with stirring at 600–800 rpm, air consumption of 0.3 ± 0.1 L/min per 5 L of medium, and pO_2_ not lower than 40%.

The dynamics of growth and the development of bacteriolytic activity under the fermentation conditions was studied (Supplementary Figure S9b). Maximum bacteriolytic activity in the culture liquid (2322 LU/mL) was observed by 30 h of fermentation. By this time, the culture growth was 4.32 at OD_540_. During the next four hours of cultivation, the growth rate did not change, and the activity did not increase.

Thus, strain *L. capsici* P_T5_-*blp* behaves stably under fermentation conditions, no lysis is observed, and bacteriolytic activity increases in proportion to the culture growth. All of this testifies to the successful scale-up of *L. capsici* P_T5_-*blp* cultivation. 

**Figure 5:**
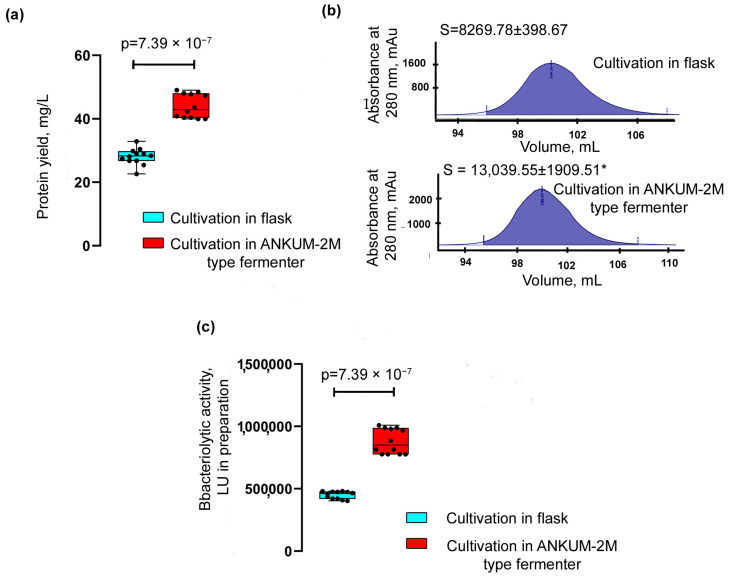
Assessment of Blp yield. (**a**) Comparison of the yield of the Blp protein of *L. capsici* P_T5_-*blp* after cultivation in a fermenter and in flasks. The boxplots show the median and IQR. The values were obtained in two independent experiments, each with six technical replicates. The two groups were compared using a two-sided Mann–Whitney *U*-test. (**b**) Peak area of the Blp protein after purification on a Hiload 16/60 column (Superdex 75). The mean values were obtained in two independent experiments, each with two technical replicates. The two groups were compared using a two-sided Mann–Whitney *U*-test (*p* = 0.0286). (**c**) Comparison of the bacteriolytic activity of the Blp of *L. capsici* P_T5_-*blp* after cultivation in a fermenter and in flasks. The boxplots show the median and IQR. The values were obtained in two independent experiments, each with six technical replicates. The two groups were compared using a two-sided Mann–Whitney *U*-test. * *p* = 0.0286.

The yield of the Blp was assessed after its purification from the culture liquid of *L. capsici* P_T5_-*blp* grown on an RM medium in flasks and in a fermenter (Figure 5a, Table 2) according to the scheme described above (Supplementary Figure S1).

The yield of Blp after cultivation in flasks was 28.057 ± 2.593 mg/L; after its cultivation in a fermenter, it was 43.991 ± 3.787 mg/L, which was 1.6 times higher.

A comparison of the areas of the Blp peaks obtained as a result of purification (Figure 5b) also showed that the protein yield during cultivation in the fermenter was 1.6 times greater. The bacteriolytic activity of the Blp preparation obtained after cultivation in a fermenter was 1.95 times higher than that of the Blp preparation after cultivation in flasks (Figure 5c, Table 2).

Thus, the successful scale-up of *L. capsici* P_T5_-*blp* cultivation increased the yield of the target Blp protein. These results point to the biotechnological potential of the developed homologous expression system.

### 2.5. The Use of the Novel Expression System for Other Bacteriolytic Enzymes of L. capsici

It was important to establish that the developed expression system can also be used for other extracellular bacteriolytic enzymes of *L. capsici* VKM B-2533^T^. To test this, we took the serine protease (Serp) of *L. capsici* VKM B-2533^T^ that we had isolated earlier [10]. This protease is capable of hydrolyzing the autoclaved *S. aureus* 209P cells and fibrin (data not shown). The recombinant strain *L. capsici* P_T5_-*serp* containing the expression vector PBBR1-MCS5 P_T5_-*serp* was obtained, in which the *serp* gene is under the regulation of the T5 promoter (Methods). The recombinant strain was cultivated in an LB-M liquid medium. After 20 h of cultivation, cell growth reached 3.16 at OD_540_; no lysis was observed.

**Figure 6:**
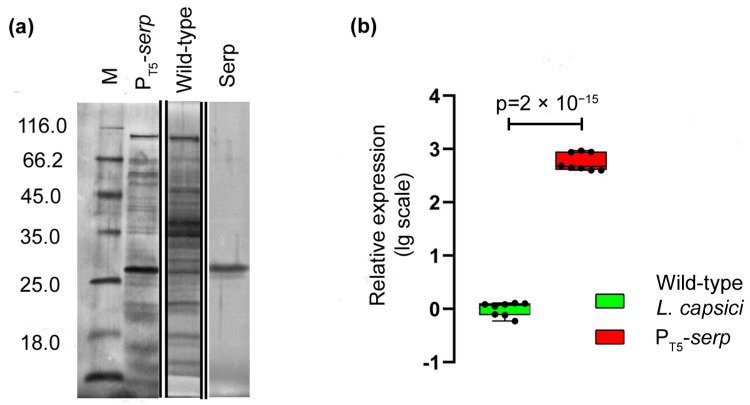
Assessment of the possibility of using the developed expression system for other *L. capsici* proteins. (**a**) SDS-PAGE: Lanes correspond to samples of culture liquid of *L. capsici* strains and purified serine protease (Serp) of *L. capsici* VKM B-2533^T^ (Supplementary Figures S10–S12). The gel was stained with imidasole–ZnCl_2_ solution. (**b**) Relative level of expression of the *serp* gene of *L. capsici serp* P_T5_ as compared with wild-type *L. capsici*. The boxplots show the median and IQR. The values were obtained in four independent experiments, each with two technical replicates. The two groups were compared using an unpaired two-tailed Student’s *t*-test, *t* = 37.41, *df* = 14.

Electrophoretic analysis demonstrated the accumulation of a protein in the culture liquid of the recombinant strain, which by its electrophoretic mobility, coincided with the serine protease Serp of *L. capsici* (Figure 6a). An analysis of the relative level of *serp* expression also showed that in *L. capsici* P_T5_-*serp* cells, it was 585 times higher than in the wild-type *L. capsici* cells (Figure 6b, Supplementary Table S4).

Thus, the results indicate the possibility of using the developed homologous expression system for other secreted enzymes of *L. capsici* VKM B-2533^T^.

## 3. Discussion

New agents are being found that show high antimicrobial efficiencies under laboratory conditions and that are practically valuable. However, the use of such agents in general practice can often be difficult or even impossible, due to the lack of, e.g., technology for their synthesis (for antibiotics), or the lack of an effective expression system for agents of a protein nature. In connection with the search for an alternative to antibiotics, protein agents, such as the bacteriolytic enzymes of bacteria, come to the fore.

Our laboratory has been studying the bacteriolytic enzymes of *Lysobacter* bacteria for many years. Many tasks that are associated with large-scale experiments on their preclinical characteristics, as well as with the design of new-generation antimicrobial drugs, are complicated by the need for the time-consuming development of material from the culture liquid of the producers. Earlier, we developed heterologous expression systems for the bacteriolytic proteases L1 and L5 of *Lysobacter* sp. XL 1 [20,21,22], as well as for *L. capsici* VKM B-2533^T^ Blp [10]. However, we encountered a number of problems related to the expression of lytic proteins in foreign cells, which led to low yields of the active protein. The idea of producing a homologous expression system arose a long time ago, but it was impossible to implement this due to a poor knowledge of the genetics of *Lysobacter* bacteria. After initial reports [23,24,25,26], we began to make progress in our experiments with regard to genetic manipulations with *Lysobacter* cells. After a successful gene knockout of the *Lysobacter* sp. XL 1 bacteriolytic enzyme L5 [27], we commenced the creation of a homologous expression system for *L. capsici* VKM B-2533^T^ Blp.

During the initial stage, it was necessary to find a plasmid maintained in *L. capsici* cells, based on which an expression vector could be constructed. No laboratory plasmid collections were maintained in the cells of this strain. We were assisted by Chinese colleagues in isolating new antibiotics in *Lysobacter* and in studying the mechanisms of their biosyntheses [26], and they kindly provided us with the plasmid pBBR1-MCS5. To date, pBBR1-MCS5 is the only known plasmid that is maintained in *Lysobacter* cells. For the next stage, it was necessary to choose a promoter among the known and studied promoters. As noted above, only two promoters are known for the bacteria of this genus, both from *L. enzymogenes*. We had not tested the promoter, which regulates the biosynthesis pathway of the antibiotic HSAF [18]. However, Wang and colleagues have shown that HSAF is inferior to the GroEL promoter in terms of efficiency [19]. For our experiments, we chose GroEL and its modification, GroEL(A), as well as the bacteriophage T5 promoter, which has not been used in *Lysobacter* cells to date. We constructed expression vectors, in which the gene of the marker fluorescent protein GFP was under the regulation of the chosen promoters, and then we developed *L. capsici*-based expression strains. All of the promoters were proven to work in the cells of the *L. capsici* expression strains. The highest fluorescence intensity was observed in *L. capsici* P_T5_-*gfp* cells, in which the GFP protein gene was under the regulation of the T5 promoter. We showed for the first time that the T5 promoter was recognized by the *L. capsici* RNA polymerase. The lowest fluorescence level was observed in *L. capsici* P_GroEL_-*gfp* cells. The next step was the construction of the expression vectors PBBR1-MCS5 P_T5_-*blp* and PBBR1-MCS5 P_GroEL(A)_-*blp*, in which the gene of the bacteriolytic protein Blp is under the regulation of the T5 and GroEL(A) promoters, respectively, and then the *L. capsici* P_T5_-*blp* and *L. capsici* P_GroEL(A)_-*blp* expression strains were obtained.

As a result of the characterization of the expression strains, it was found that in *L. capsici* P_GroEL(A)_-*blp* and *L. capsici* P_T5_-*blp*, the expression levels of the *blp* gene were 246- and 667-fold higher, respectively, as compared with the wild-type strain. Thus, the gene of the Blp protein was successfully expressed under the regulation of the GroEL(A) and the T5 promoters. Herewith, there was an increase in the bacteriolytic activity of the culture liquid of the expression strains with respect to live *S. aureus* 209 P cells. The yield of Blp in *L. capsici* P_GroEL(A)_-*blp* was 13.755 mg/L, and in *L. capsici* P_T5_-*blp*, it was 17.519 mg/L, which was 6.7- and 8.5-fold greater, respectively, compared with wild-type *L. capsici.* The bacteriolytic activities of the produced homogeneous Blp preparations from the *L. capsici* P_GroEL(A)_-*blp* and *L. capsici* P_T5_-*blp* strains were 7.8- and 10.5-fold higher, respectively, as compared with the Blp preparation obtained from the wild-type strain. Thus, we developed successful homologous expression systems for the bacteriolytic enzyme Blp, enabling an increase of the yield of this practicably valuable protein. The cells of both expression strains are characterized by stable growth and Blp production. Herewith, the strain *L. capsici* P_T5_-*blp* was reliably established to be a more effective producer of Blp, as compared with *L. capsici* P_GroEL(A)_-*blp*. 

Some features of the developed expression systems were also noted. Thus, a change in the profiles of major proteins of the culture liquid was observed in the expression strains. There was also some accumulation of protein in the cells. This could be due to a change in the regulation of the genes of the secreted proteins, or else the secretory apparatus could not cope with the increased load. All of this requires further study. Herewith, there are visible prospects for improving the developed expression system. Examples include the genetically mediated optimization of the secretory apparatus, the deletion of the ballast protein genes, or for that matter, the use of the CRISPR/dCas9 system to enhance the expression of target products. Such a system has already been used to enhance the production of cyclic lipodepsipeptide antibiotics WAP-8294A in *L. enzymogenes* OH11 [28]. It will be of interest to subsequently apply this approach for the homologous expression system that we have developed. 

For the next stage, we successfully upscaled the cultivation of the expression strain *L. capsici* P_T5_-*blp* in a 10 L fermenter. For this process, we used a previously developed nutrient medium and previous significant experience on the cultivation of *Lysobacter* sp. XL1. The growth of the culture under fermentation conditions and the production of Blp were stable. A particular success was the increase in Blp yield, which amounted to 43.991 ± 3.787 mg/L, which was 1.6-fold higher than the yield of this protein when cultivated in flasks.

As we have already noted, it is difficult to create successful expression systems for lytic enzymes. The idea was based on the creation of an expression strain that was based on a well-known producer of bacteriolytic enzymes. The difficulty of this was that bacteria of this genus are almost never used for expression cultures. There is work in which the cells of *L. enzymogenes* OH11 have been used as recombinant strains for expressing the gene of the quorum-quenching enzyme of *Muricauda olearia* Th120 [19]. It was this group of researchers who previously used the GroEL(A) promoter. They also showed that during cultivation, the recombinant strain eliminates the plasmid, which was one of the reasons for why it could not be used for practical applications. We note here that we did not have such a problem.

Thus, we developed the first successful, scalable expression system for a bacteriolytic enzyme, Blp, of *L. capsici* VKM B-2533^T^. Special value lies in the fact that this system yields bacteriolytic enzymes produced by Gram-negative bacteria. Until recently, successful expression systems were known only for bacteriolytic enzymes produced by Gram-positive bacteria—enterolysin A of *Enterococcus faecalis* II/1, based on *Escherichia coli* SG13009 [8], and zoocin A of *Streptococcus equi* subsp. *zooepidemicus* 4881, based on *E. coli* M15 [29], enabled the production of proteins at rates of 20 mg/L and 30 mg/L, respectively. Expression systems based on the cells of *E. coli* TOP10 [30] and *Pichia pastoris* GS115 [31] make it possible to produce recombinant lysostaphin at rates of 200 mg/L and 250 mg/L, respectively. We should also mention a recently published work on the development of a heterologous expression system for *L. enzymogenes* M497-1 Blp by using *B. subtilis* 168 as an expression strain [32]. However, it is difficult to assess the biotechnological significance of the developed expression system from the results of the work, due to its methodological inaccuracies and its lack of statistics. 

In conclusion, it should be noted that we observed significant biotechnological potential for the developed homologous expression system. The system is constitutive, i.e., it requires no inducer in the cultivation medium. This is an extra advantage for the biotechnological aspect of its production. This system reacts quite easily to additional modifications. For example, it can be used to express other secreted *L. capsici* enzymes, which was exemplified in this article by the bacteriolytic serine protease Serp. We have already mentioned the prospects for optimizing the secretory process. Currently, work is already underway to optimize the Blp purification scheme by adding a His-tag to the C-end of the protein. All of our results allow us to hope for obtaining a highly effective expression system for bacteriolytic enzymes of Gram-negative bacteria in the near future, based on the system we have developed and that we present in this article.

## 4. Materials and Methods

### 4.1. Bacterial Strains, Plasmids, and Cultivation Conditions

The bacterial strains and plasmids used are listed in Table 3. 

The *E. coli* strain XL1-Blue was grown on LB medium (g/L): tryptone, 10; yeast extract, 5; NaCl, 10; and pH 7.0 at 37 °C. All *L. capsici* strains were cultivated at 29 °C with stirring (205 rpm) on a PSU-20i orbital shaker (Biosan, Rīga, Latvia) on media of the following composition (g/L): modified LB medium (LB-M): peptone, 5; yeast extract, 5; NaCl, 5; pH 7.5 [39]; RM: glucose, 5.0; peptone, 2.0; yeast extract, 2.0; Na_2_HPO_4_·12H_2_O, 4.2; KH_2_PO_4_, 1.0; KCl, 0.6; MgSO_4_·7H_2_O, 5.0; FeSO_4_·7H_2_O, 0.1; and pH 7.0 [13]. The corresponding antibiotics were added to liquid and agarized media in the following concentrations (µg/mL): Gm for *E. coli*, 10; and Gm for *L. capsici* expression strains, 20.

### 4.2. Molecular Genetic Kits and Equipment

All molecular genetic procedures were performed in accordance with the recommendations of the kits’ manufacturers, and Sambrook and Russell’s manual [40]. Restriction endonucleases, alkaline phosphatase, T4 DNA ligase, and T4 polynucleotide kinase were used (Thermo Fisher Scientific, Waltham, MA, USA). The PCR analysis was performed using Q5 DNA polymerase (New England Biolabs, Ipswich, MA, USA) on a Mastercycler ProS amplifier (Eppendorf, Hamburg, Germany). The entire list of oligonucleotides is provided in Supplementary Table S5. The PCR reactions (total volume, 50 µL) were conducted under the following conditions: 200 mM dNTP, 0.5 µM forward and reverse primers, DNA template (Supplementary Table S6), 0.02 U/µL Q5 high-fidelity DNA polymerase (New England Biolabs, Ipswich, MA, USA) in 1× reaction buffer containing 2 mM MgCl_2_. The thermal cycles were programmed according to the manufacturer’s protocol: Initial denaturation at 98 °C for 30 s, followed by 20–30 cycles of 98 °C for 10 s, an annealing temperature of 60 °C for 20 s, 72 °C for a length of time that was determined from the amplicon length (extension times were 30 s per kb), and a final extension at 72 °C for 2 min.

DNA electrophoresis was performed in 0.8% agarose gel based on agarose (Merck, Darmstadt, Germany) in a TAE buffer containing 0.5 mg/mL of ethidium bromide. DNA visualization in the gel was conducted on a Bio-Print ST4 instrument (Vilber Lourmat, Collégien, France) at 354 nm. To isolate the DNA from the gel, the QIAquick gel extraction kit (Qiagen, Germantown, MD, USA) was used. A quantum prep plasmid miniprep kit (Bio-Rad, Hercules, CA, USA) was used to isolate plasmids from *E. coli* XL1-Blue. A QIAamp DNA mini kit (Qiagen, Germantown, MD, USA) was used to isolate genomic DNA from *L. enzymogenes* VKM B-2235^T^ and *L. capsici* VKM B-2533^T^. The quality of the DNA preparations was checked, and a quantitative analysis was carried out electrophoretically in 0.8% agarose gel and on a NanoPhotometer P360 instrument (IMLEN, Schatzbogen, Germany). The electroporation of all constructed plasmids (Table 1) in *L. capsici* VKM B-2533^T^ was performed on a MicroPulser electroporator (Bio-Rad, Hercules, CA, USA). 

### 4.3. Chemical Transformation and Electroporation of Plasmids into Bacterial Cells

Highly competent *E. coli* XL1-Blue cells were transformed using a ligation mixture using the RbCl method [41]. The electroporation of *L. capsici* VKM B-2533^T^ using constructed plasmids (Table 3) was conducted in accordance with the method of Lin, with a modification [27]. Briefly, a 20 µL volume of competent *L. capsici* cells (in 10% ice-cold glycerol) was mixed with 500 ng of constructed plasmids and transferred to 0.2 cm electroporation cuvettes (Bio-Rad, USA). Electroporation was performed using a Gene Pulser apparatus (Bio-Rad, Hercules, CA, USA) at 12.5 kV/cm. After electroporation, 1 mL of a sterile LB-M was added to the cells, which were then incubated at 29 °C for 3 h without stirring. The cells were then cultivated on an agarized LB-M with the antibiotic Gm.

### 4.4. Construction of Plasmids to Obtain L. capsici Expression Strains

PBBR1-MCS5 P_T5_-*gfp*: The amplicon obtained as a result of PCR with the specific primers T5_KpnI (forward) and T5_XbaI (reverse) to a fragment containing T5 promoter–*gfp*–terminator lambda t0 from the pTurboGFP-B plasmid (amplicon size, 1063 bp) was ligated into a PBBR1-MCS5 plasmid treated using the KpnI/XbaI restriction sites. The ligation mixture was used to transform *E. coli* XL1-Blue cells. The clones were selected on an agarized LB medium with Gm. Electroporation of the isolated plasmid PBBR1-MCS5 P_T5_-*gfp* into competent *L. capsici* cells was performed according to the procedure described above. The selection of *L. capsici* P_T5_-*gfp* clones was performed on an agarized LB-M medium with Gm.

PBBR1-MCS5 P_GroEL_-*gfp*: The GroEL promoter, amplified with the genomic DNA of *L. enzymogenes* VKM B-2235^T^ using the specific primers Gro_KpnI (forward) and Gro_HindIII (reverse) (amplicon size, 199 bp), the *gfp* gene amplified from the plasmid pTurboGFP-B using the specific primers GFP_HindIII (forward) and GFP_BamHI (reverse) (amplicon size, 705 bp), and the *rrnB* gene containing two terminator regions T1 and T2, amplified from the plasmid pEX18 using the specific primers Term_BamHI (forward) and Term_XbaI (reverse) (amplicon size, 230 bp), were sequentially cloned into the PBBR1-MCS5 plasmid. At each stage, *E. coli* XL1-Blue clones were selected on an agarized LB medium with Gm. The electroporation of the isolated plasmid PBBR1-MCS5 P_GroEL_-*gfp* into competent *L. capsici* cells was performed according to the above-described procedure. Clones of *L. capsici* P_GroEL_-*gfp* were selected on an agarized LB-M medium with Gm.

PBBR1-MCS5 P_GroEL(A)_-*gfp*: The amplicon obtained from the PCR with the specific primers Gro_KpnI (forward) and Gro(A)_HindIII (reverse) to the GroEL promoter from the PBBR1-MCS5 P_GroEL_-*gfp* plasmid (amplicon size, 200 bp) was ligated into the PBBR1-MCS5 P_GroEL_-*gfp* plasmid treated using the KpnI/HindIII restriction sites. The GroEL(A) and GroEL promoters differ in a modification of the distance from the start codon to the Shine–Dalgarno region by adding the nucleotide A before the start codon [19]. Electroporation of the isolated plasmid PBBR1-MCS5 P_GroEL(A)_-*gfp* into *L. capsici* competent cells was performed according to the above-described method. Clones of *L. capsici* P_GroEL(A)_-*gfp* were selected on an agarized LB-M medium with Gm.

PBBR1-MCS5 P_GroEL(A)_-*blp*: The amplicon obtained as a result of the PCR with the specific primers Blp1_HindIII (forward) and BlpI_BamHI (reverse) to the *blp* gene with the genomic DNA of *L. capsici* VKM B-2533^T^ (amplicon size, 1143 bp) was ligated into the plasmid PBBR1-MCS5 P_GroEL(A)_-*gfp* treated using the BamHI/HindIII restriction sites. The ligation mixture was used to transform *E. coli* XL1-Blue cells. Electroporation of the isolated plasmid PBBR1-MCS5 P_GroEL(A)_-*blp* into *L. capsici* competent cells was performed according to the above-described procedure. Clones of *L. capsici* P_GroEL(A)_-*blp* were selected on an agarized LB-M medium with Gm.

PBBR1-MCS5 P_T5_-*blp*: the amplicon obtained as a result of PCR with the specific primers Blp2_BamHI (forward) and Blp2_HindIII (reverse) to the *blp* gene from the plasmid PBBR1-MCS5 P_GroEL(A)_-*blp* (amplicon size, 1143 bp) was ligated into the plasmid PBBR1-MCS5 P_T5_-*gfp* treated using the BamHI/HindIII restriction sites. The ligation mixture was used to transform *E. coli* XL1-Blue cells. Electroporation of the isolated plasmid PBBR1-MCS5 P_T5_-*blp* into competent *L. capsici* cells was carried out according to the above-described procedure. Clones of *L. capsici* P_T5_-*blp* were selected on an agarized LB-M medium with Gm. 

PBBR1-MCS5 P_T5_-*serp*: the amplicon obtained as a result of the PCR with specific primers Serp_BamHI (forward) and Serp_HindIII (reverse) to the *serp* gene with the genomic DNA of *L. capsici* VKM B-2533^T^ (amplicon size, 1383 bp) was ligated into the plasmid PBBR1-MCS5 P_T5_-*gfp* treated using the BamHI/HindIII restriction sites. The ligation mixture was used to transform *E. coli* XL1-Blue cells. Electroporation of the isolated plasmid PBBR1-MCS5 P_T5_-*serp* into competent *L. capsici* cells was performed according to the above-described procedure. Clones of *L. capsici* P_T5_-*serp* were selected on an agarized LB-M medium with Gm.

The correctness of the assembled constructions was confirmed using PCR with specific oligonucleotides (Supplementary Table S5) and from sequencing performed at Evrogen CJSC (Moscow, Russia). 

### 4.5. Selection of Promoter for Creating a Homologous Expression System Based on L. capsici

Promoters were screened using GFP. The strains *L. capsici* VKM B-2533^T^, *L. capsici* P_GroEL_-*gfp*, *L. capsici* P_GroEL(A)_-*gfp*, and *L. capsici* P_T5_-*gfp* were cultivated in an LB-M liquid medium for 18 h at 29 °C with stirring at 205 rpm. Cells aligned in optical density to OD540 = 3.7 were centrifuged at 12,000× *g* for 10 min, dissolved in 500 µL of 10-mM Tris, pH 8.0, and disintegrated via sonication (amplitude 3, medium mode). The cell debris was then removed via centrifugation at 12,000× *g*, 10 min, and 200 µL of the supernatant was sampled for analysis. Fluorescence was measured on a FilterMax F5 microplate reader (Molecular Devices, San Jose, CA, USA) at an excitation wavelength of 485 nm and an emission wavelength of 535 nm.

### 4.6. Gene Expression Analysis Using RT-qPCR

The strains *L. capsici* VKM B-2533^T^, *L. capsici* P_GroEL(A)_-*blp*_,_ *L. capsici* P_T5_-*blp*, and *L. capsici* P_T5_-*serp* were cultivated on an LB-M medium for 18 h at 29 °C with stirring at 205 rpm. The total bacterial RNA was isolated with an Aurum Total RNA Mini Kit (Bio-Rad, Hercules, CA, USA) from cells aligned in optical density to OD540 = 3.0, in accordance with the manufacturer’s recommendation. The DNA was removed on columns treated with DNase (Bio-Rad, Hercules, CA, USA). The obtained preparations of total bacterial RNA were analyzed on a P360 NanoPhotometer (IMLEN, Schatzbogen, Germany) to determine the concentration of samples and the A260/A280 index, and electrophoretically separated in 4% PAG with 8 M urea (400 ng of each preparation was applied) (Supplementary Figure S13). The oligonucleotides were designed using the Primer-BLAST program (https://www.ncbi.nlm.nih.gov/tools/primer-blast/ accessed on 2 February 2021) (Supplementary Table S7). 

For all pairs of oligonucleotides, the amplification efficiency was determined from the calibration curve constructed for a series of DNA dilutions. The specificity of the reaction was confirmed via electrophoresis in a 0.8% agarose gel. The cDNA synthesis (474 ng RNA was taken for the reaction) was carried out using a RevertAid RT Reverse Transcription Kit (Thermo Fisher Scientific, Waltham, MA, USA), according to the manufacturer’s instructions, and with specific oligonucleotides. All qRT-PCR reactions were performed using a set with SynTag DNA polymerase with enzyme activity-inhibiting antibodies in the presence of SYBR Green I (Syntol, Moscow, Russia) on a DTlite instrument (DNA Technology, Moscow, Russia). qRT-PCR consisted of 40 cycles (95 °C for 20 s, 60 °C for 20 s, and 72 °C for 5 s). The crossing points (Cp) were extracted with RealTime_PCR_A7A514 software. The relative level of expression was determined using the comparative quantitative assessment method (E^−ΔΔCp^) [42], based on four independent repeats for each sample, using *L. capsici* VKM B-2533^T^ as a control sample according to the formula: E_gene of interest_ (Cp control gene of interest − Cp experimental gene of interest)/E_reference gene_ (Cp control reference gene − Cp experimental reference gene).

To validate the method, the *gntR* gene was used as a reference gene; its stability was determined using the Best Keeper program [43]—SD 0.74 and CV 3.76%. The RT-qPCR reactions were conducted for two independent experiments.

### 4.7. Blp Purification

Blp from the culture liquid of strains *L. capsici* VKM B-2533^T^, *L. capsici* P_GroEL(A)_-*blp*, and *L. capsici* P_T5_-*blp* was purified according to the scheme that we previously developed [10]. The strains were cultivated in 0.45 L LB-M liquid medium with addition of the antibiotic Gm for 20 h at 29 °C, with stirring at 205 rpm. The culture liquid was then released from the cells at 5000× *g* for 30 min at 4 °C in an Avanti J-26XP centrifuge (Beckman, Brea, CA, USA). Proteins were precipitated from equal volumes of culture liquid (395 mL) by adding ammonium sulfate (NH_4_)_2_SO_4_ to 80% saturation and with subsequent centrifugation at 25,960× *g* for 60 min. The resulting precipitates were dissolved in 50 mM Tris-HCl, pH 8.0, dialyzed against the same buffer, and further purified using the following column chromatography methods: cation exchange chromatography using a Toyopearl CM-650 carrier column (Merck, Darmstadt, Germany), cation exchange chromatography using an EnrichS column (Bio-Rad, Hercules, CA, USA), and gel filtration on the Hiload 16/60 (Superdex 75) column (Amersham Biosciences, Uppsala, Sweden) using an NGC chromatographic system (Bio-Rad, Hercules, CA, USA). All fractions were analyzed during the purification process—the total bacteriolytic activity and the total protein concentration were determined. The 24 peak fractions containing the homogeneous enzyme Blp obtained after gel filtration were combined. The homogeneity of the obtained Blp preparations was confirmed electrophoretically and via the MALDI-TOF method. The purification scheme is shown in the Supplementary Figure S1.

The peak areas obtained after Blp purification from each strain were determined using ChromLab 3.3.0.09 software (24 homogeneous enzyme fractions, which were then combined, were used to determine the peak area).

### 4.8. MALDI-TOF Mass Spectrometry

MALDI-TOF was performed in accordance with the previously described method [10].

### 4.9. SDS-PAGE

Protein electrophoresis was performed in 12.5% PAG in the presence of SDS via the Laemmli method [44]. The preparations were aligned by volume (12 µL) in the case of the protein fractions and culture liquids, or by optical density in the case of the cells (10 OD units/mL). The samples were heated in sample buffer at 99 °C for 10 min. A mixture of protein standards (Thermo Fisher Scientific, Waltham, MA, USA) was used as markers: β-galactosidase, 116.0 kDa; BSA, 66.2 kDa; ovalbumin, 45.0 kDa; lactate dehydrogenase, 35.0 kDa; REase Bsp981, 25.0 kDa; β-lactoglobulin, 18.4 kDa; and lysozyme, 14.4 kDa. Electrophoresis in a concentrating gel was performed at 90 V, and in a separating gel, at 180 V. Protein bands in the gel were detected using imidazole staining and ZnCl_2_ solutions [45], or Coomassie Brilliant Blue R-250 (Serva, Heidelberg, Germany).

### 4.10. RNA Polyacrylamide Gel Electrophoresis

Electrophoresis of the RNA of *L. capsici* VKM B-2533^T^, *L. capsici* P_GroEL(A)_-*blp*, *L. capsici* P_T5_-*blp*, and *L. capsici* P_T5_-*serp* was performed in 4% PAG in TBE buffer containing 8 M urea [46]. RNA preparations (400 ng each) in formamide gel loading buffer (47.5% formamide, 2.5 mM EDTA, and 0.013% bromophenol blue pH 8.0), heated at 90 °C for 7 min, were injected into the gel. Electrophoresis was performed at 60 V in TBE buffer. The RNA was stained by soaking the gel in TBE buffer containing 1 µg/mL of ethidium bromide for 10 min, followed by detection using a P360 Nanophotometer (IMLEN, Schatzbogen, Germany) at 280 nm.

### 4.11. Determination of Protein Concentration

The total protein concentration in the preparations was measured using the Bradford method [47]. To set up the reaction, a protocol for the microplates proposed for the proprietary reagent, Coomassie (Thermo Scientific, Waltham, MA, USA), was used. Absorption was measured at 595 nm on an iMark Microplate Absorption Reader enzyme immunoassay photometer (Bio-Rad, Hercules, CA, USA). The protein concentration was determined with the calibration curve of concentration vs. absorption, constructed for an aqueous solution of BSA (Sigma, Ronkonkoma, NY, USA) within the range of 1–25 µg/mL.

### 4.12. Determination of Bacteriolytic Activity

The bacteriolytic activity in the culture liquid of the strains and the fractions obtained during purification was determined using the turbidimetric method. Live *S. aureus* 209P cells were used as a substrate. A 5 µL preparation was added to a 0.995 mL suspension of live *S. aureus* 209 P cells in 10 mM Tris-HCl, pH 8.0, with an absorption of 0.5 at 540 nm. The mixture was incubated at 37 °C for 5 min. The control was 10 mM Tris-HCl, pH 8.0. The reaction was stopped by placing the test tubes on ice. A decrease in the absorption of the suspension was recorded in the samples at 540 nm, on a DU 730 spectrophotometer (Beckman, Brea, CA, USA).

LUs were calculated via the following formula: {[0.5 (initial OD540 of the suspension) − final OD540] × 1000 µL (total reaction volume)}/[5 min (time of reaction) × 25 µL (volume of sample) × 0.01 (correction coefficient for the OD reduction per min)].

The specific activity of the purified Blp preparations was determined as a ratio of lytic activity (LU) per mg protein. 

### 4.13. Statistical Analysis

Statistical analysis was performed using GraphPad Prism version 8.0.1 (GraphPad Software, San Diego, CA, USA). All experiments were conducted with at least three repeats. The data are presented as means ± standard deviations, as well as in the form of boxplots (medians ± interquartile spans). The data were considered to be significant at *p* < 0.05. The normal distribution of the data was verified using the D’Agostino–Pearson complex test. To determine the equality of the variances of two independent groups, the *F*-test was used; when comparing the variances of more than two groups, the Brown–Forsythe test was used. For the normally distributed data of two groups, the two-sided unpaired Student’s *t*-test was used; for other data types, the two-sided Mann–Whitney *U*-test was used. To compare more than two groups with a normal distribution and equal variances, a one-sided analysis of variance (ANOVA) was used, followed by the Tukey test for multiple comparison; for unequal variances, the Welch’s ANOVA test with the Tamhane T2 test for multiple comparison was used.

## Figures and Tables

**Table 1:** Bacteriolytic activity of the culture liquid of *L. capsici* strains.

Strains	LU/mL
*L. capsici* VKM B-2533^T^	266 ± 26
*L. capsici* P_GroEL(A)_-*blp*	802 ± 270
*L. capsici* P_T5_-*blp*	1026 ± 262

The results are shown as means ± standard deviations. The mean values were obtained in two independent experiments, each with two technical replicates. The two groups were compared using a two-sided Mann–Whitney *U*-test (*p* = 0.0286).

**Table 2:** Comparative characterization of Blp homogeneous preparations.

Blp Preparation	Bacteriolytic Activity, LU in Preparation	Blp Yield, mg/L
Purification of Blp from culture liquid of *L. capsici* strains after cultivation on LB-M medium, 29 °C, 20 h		
Blp preparation from *L. capsici* VKM B-2533^T^	27,479 ± 888	2.061 ± 0.110
Blp preparation from *L. capsici* P_GroEL(A)_-*blp*	214,140 ± 21,146	13.755 ± 1.254
Blp preparation from *L. capsici* P_T5_-*blp*	288,200 ± 20,484	17.519 ± 2.017
Purification of Blp from culture liquid of *L. capsici* P_T5_-*blp* after cultivation in flasks and in ANKUM-2M fermenter on RM medium, 29 °C, 30 h		
Blp preparation from *L. capsici* P_T5_-*blp* at cultivation in flasks	449,840 ± 29,859	28.057 ± 2.593
Blp preparation from *L. capsici* P_T5_-*blp* at cultivation in fermenter	879,147 ± 99,186 ***	43.991 ± 3.787 ***

The results are shown as means ± standard deviations. The mean values were obtained in two independent experiments, each with six technical replicates. The two groups were compared using a two-sided Mann–Whitney U-test, *** *p* = 7.39 × 10^−7^.

**Table 3:** Strains and plasmids used in the work.

Strains and Plasmids	Characteristics	Ref.
*L. capsici* VKM B-2533^T^	Wild type	[33]
*L. enzymogenes* VKM B-2235^T^	Wild type	[34]
*L. capsici* P_GroEL_-*gfp*	Strain *L. capsici* VKM B-2533^T^ with plasmid PBBR1-MCS5 P_GroEL_-*gfp*	This work
*L. capsici* P_GroEL(A)_-*gfp*	Strain *L. capsici* VKM B-2533^T^ with plasmid PBBR1-MCS5 P_GroEL(A)_-*gfp*	This work
*L. capsici* P_T5_-*gfp*	Strain *L. capsici* VKM B-2533^T^ with plasmid PBBR1-MCS5 P_T5_-*gfp*	This work
*L. capsici* P_GroEL(A)_-*blp*	Strain *L. capsici* VKM B-2533^T^ with plasmid PBBR1-MCS5 P_GroEL(A)_-*blp*	This work
*L. capsici* P_T5_-*blp*	Strain *L. capsici* VKM B-2533^T^ with plasmid PBBR1-MCS5 P_T5_-*blp*	This work
*L. capsici* P_T5_-*serp*	Strain *L. capsici* VKM B-2533^T^ with plasmid PBBR1-MCS5 P_T5_-*serp*	This work
*E. coli* XL1–Blue	*recA1 endA1 gyrA96 thi hsdR17 supE44 relA1 lac*/[F′::Tn10 *proAB + lacI^q^ lacZΔM15 traD36*]	[35]
pBBR1-MCS5 *	Vector with P_Lac_-promoter, Gm^R^	[36]
pTurboGFP-B	Template for amplification of fragment T5 promoter—*gfp*—terminator lambda t0	[37]
pEX18	Template for amplification of terminators T1 and T2	[38]
PBBR1-MCS5 P_GroEL_-*gfp*	Plasmid pBBR1-MCS5 with *gfp* gene under the regulation of GroEL promoter	This work
PBBR1-MCS5 P_GroEL(A)_-*gfp*	Plasmid pBBR1-MCS5 with *gfp* gene under the regulation of GroEL(A) promoter	This work
PBBR1-MCS5 P_T5_-*gfp*	Plasmid pBBR1-MCS5 with *gfp* gene under the regulation of T5 promoter	This work
PBBR1-MCS5 P_GroEL(A)_-*blp*	Plasmid pBBR1-MCS5 with *blp* gene under the regulation of GroEL(A) promoter	This work
PBBR1-MCS5 P_T5_-*blp*	Plasmid pBBR1-MCS5 with *blp* gene under the regulation of T5 promoter	This work
PBBR1-MCS5 P_T5_-*serp*	Plasmid pBBR1-MCS5 with *serp* gene under the regulation of T5 promoter	This work

* The plasmid was a kind gift of Dr. Yangyang Zhao (Institute of Plant Protection, Jiangsu Academy of Agricultural Sciences, Jiangsu Key Laboratory for Food Quality and Safety—State Key Laboratory Cultivation Base of Ministry of Science and Technology, Nanjing, China).

## Data Availability

The whole-genome shotgun project of *L. capsici* VKM B-2533^T^ has been deposited in DDBJ/ENA/GenBank under accession no. CP094357.1. The Serp, Blp, and GntR proteins have the locus tags IEQ11_RS08595 (protein_id=WP_191822808.1), IEQ11_RS04180 (protein_id=WP_191821694.1) and IEQ11_RS12995 (protein_id=WP_036104172.1), respectively. All other relevant data are available from the corresponding author upon request.

## Supplementary Materials

The following supporting information can be downloaded at: https://www.mdpi.com/article/10.3390/ijms23105722/s1.
