# Supplementary material for: The First Homologous Expression System for the β-Lytic Protease of Lysobacter capsici VKM B-2533T, a Promising Antimicrobial Agent
Source: Int J Mol Sci. 2022 May 20;23(10):5722. doi: 10.3390/ijms23105722 (PMC9145596; doi:10.3390/ijms23105722)
Supplement: Supplementary file 1 [file ijms-23-05722-s001.zip › ijms-1710407-supplementary.pdf]

**Supplementary Table S1.** Fluorescence intensity in cells of *L. capsici* strains.

| ***L. capsici* strains** | **Fluorescence intensity, arbitrary units x $10^7$** |
|---|---|
| Wild-type *L. capsici* VKM B-2533$^T$ | 0.083±0.011 |
| *L. capsici* $P_{GroEL}$-*gfp* | 1.557±0.101 |
| *L. capsici* $P_{GroEL(A)}$-*gfp* | 3.625±0.377 |
| *L. capsici* $P_{T5}$-*gfp* | 46.750±3.366 |

Results are shown as means ± standard deviations. The mean values were obtained in three independent experiments: two independent experiments with two technical replicates and one independent experiment with six technical replicates.

**Supplementary Table S2.** Relative level of expression of the *blp* gene in cells of *L. capsici* strains.

| ***L. capsici* strains** | **Relative level of expression of the *blp* gene** |
|---|---|
| *L. capsici* VKM B-2533$^T$ | 1.02±0.21 |
| *L. capsici* $P_{GroEL(A)}$-*blp* | 250.59±82.42 |
| *L. capsici* $P_{T5}$-*blp* | 679.94±271.38 |

Results are shown as means ± standard deviations.
The mean values were obtained in four independent experiments, each with two technical replicates.

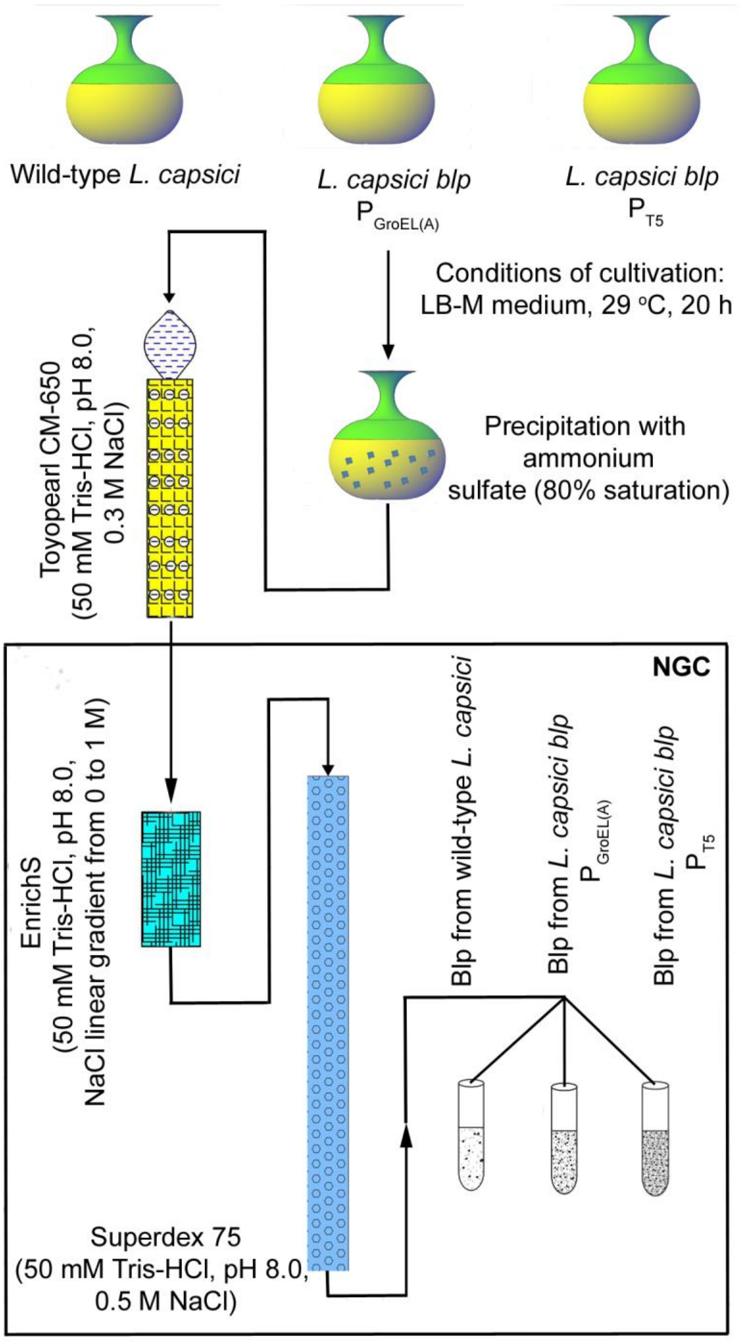


**Supplementary Figure S1**. Scheme of Blp protein purification from the culture liquid of *L. capsici* strains.

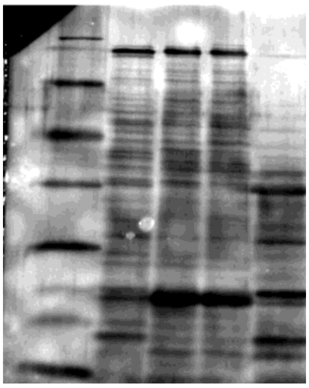


**Supplementary Figure S2.** Original gel image for Figure 3a: lanes 1, 2, 3 correspond to samples of culture liquid of *L. capsici* strains of Figure 3a.

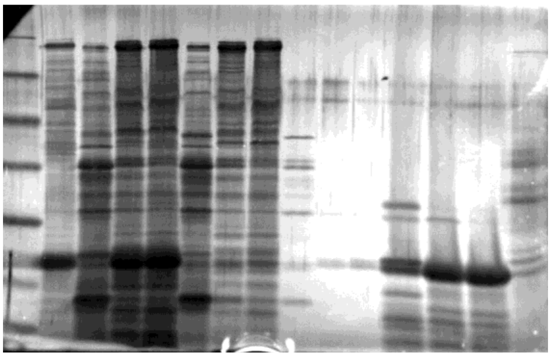


**Supplementary Figure S3.** Original gel images for Figure 3a: lane 1 corresponds to M of Figure 3a; lanes 2, 3, 4 correspond to samples after ammonium sulfate precipitation of Figure 3a; lanes 5, 6, 7 correspond to samples after Toyopearl CM-650 of Figure 3a.

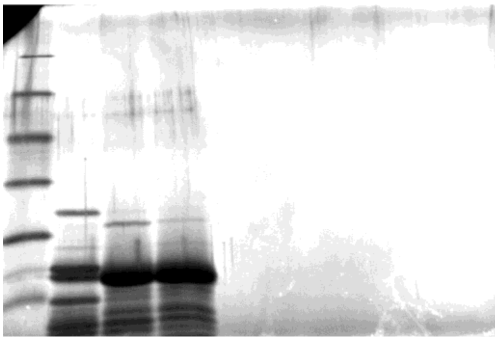


**Supplementary Figure S4.** Original gel images for Figure 3a: lanes 1, 2, 3 correspond to samples after EnrichS of Figure 3a.

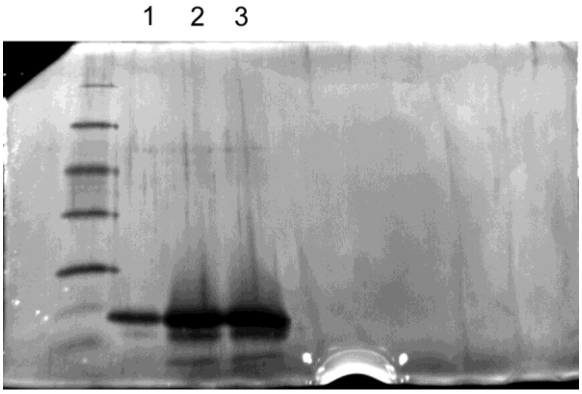


**Supplementary Figure S5.** Original gel images for Figure 3a: lanes 1, 2, 3 correspond to samples after Superdex75 of Figure 3a.

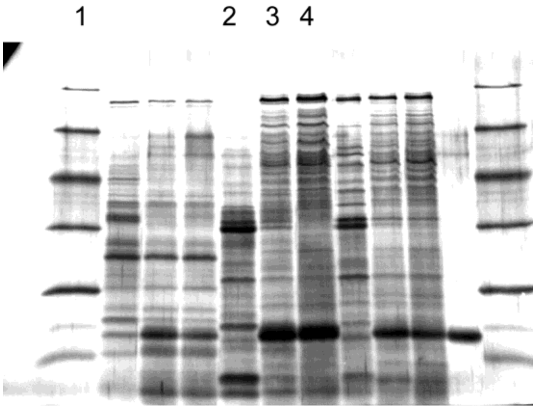


**Supplementary Figure S6.** Original gel images for Figure 4a: lane 1 corresponds to M of Figure 4a; lanes 2, 3, 4 correspond to samples of culture liquid of *L. capsici* strains of Figure 4a.

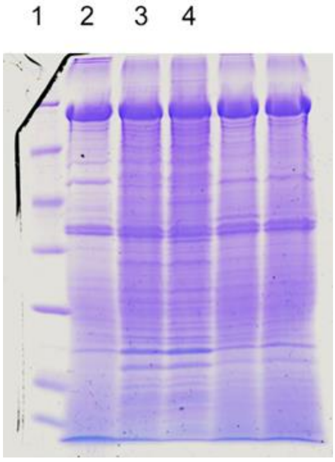


**Supplementary Figure S7.** Original gel images for Figure 4b: lane 1 corresponds to M of Figure 4b; lines 2, 3, 4 correspond to samples of *L. capsici* strains cells of Figure 4b.

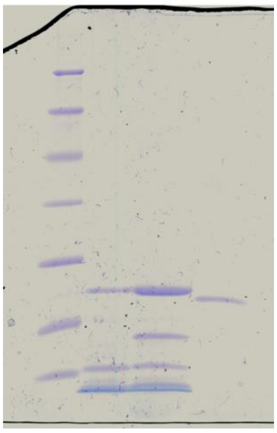


**Supplementary Figure S8.** Original gel images for Figure 4b: lane 1 corresponds to sample of purified Blp of *L. capsici* VKM B-2533$^T$ strain of Figure 4b.

**Supplementary Table S3**. Relative level of expression of the *alpA* gene.

| *L. capsici* strains | Relative level of expression of the *alpA* gene |
|---|---|
| *L. capsici* VKM B-2533$^T$ | 1.04±0.30 |
| *L. capsici* P$_{T5}$-*blp* | 0.17±0.09*** |

Results are shown as means ± standard deviations. The mean values were obtained in four independent experiments, each with two technical replicates. The two groups were compared using Student's *t*-test, $p = 8.014 \times 10^{-9}$.

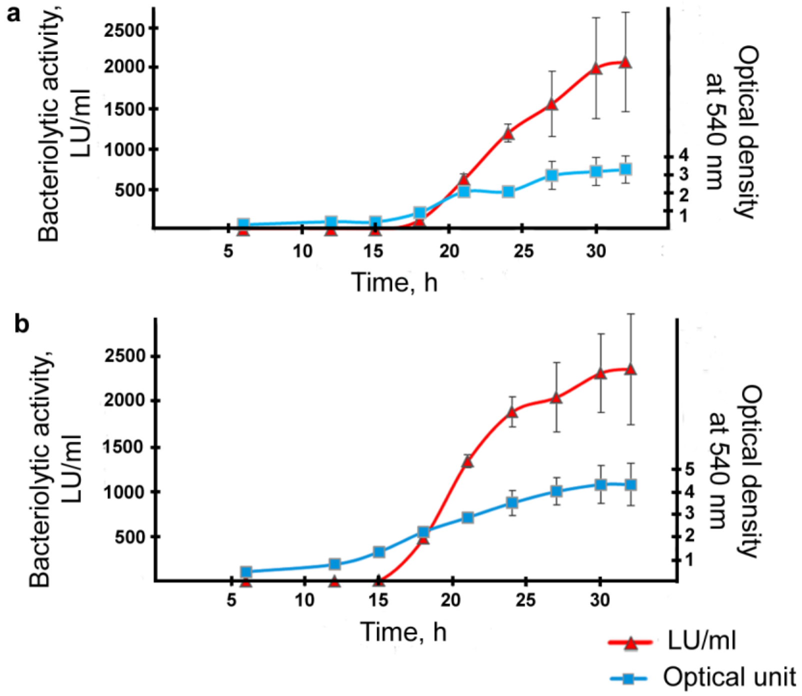


**Supplementary Figure S9.** Dynamics of growth and development of the bacteriolytic activity of *L. capsici* P$_{T5}$-*blp* strain at the cultivation on RM medium. **(a)** Cultivation in flask. **(b)** Cultivation in ANKUM-2M fermenter.

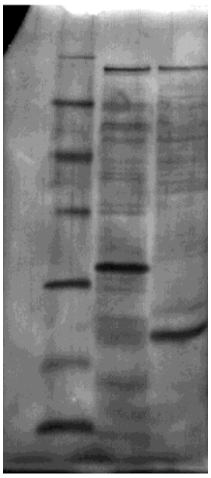


**Supplementary Figure S10.** Original gel images for Figure 6a: lane 1 corresponds to M of Figure 6a; lane 2 corresponds to sample of culture liquid of *L. capsici* $P_{T5}$-*serp* strain of Figure 6a.

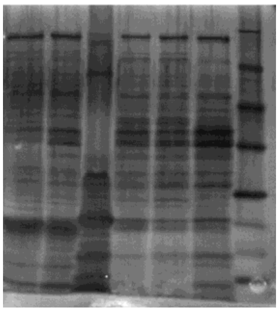


**Supplementary Figure S11.** Original gel images for Figure 6a: lane 1 corresponds to sample of culture liquid of wild-type *L. capsici* strain of Figure 6a.

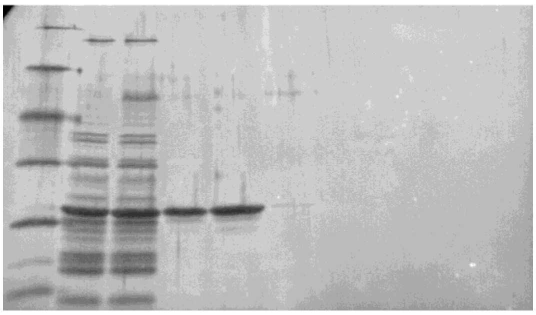


**Supplementary Figure S12.** Original gel images for Figure 6a: lane 1 corresponds to sample of purified Serp of *L. capsici* VKM B-2533$^T$ strain of Figure 6a.

**Supplementary Table S4**. Relative level of expression of the *serp* gene.

| *L. capsici* strains | Relative level of expression of the *serp* gene |
|---|---|
| *L. capsici* VKM B-2533$^T$ | 1.04±0.27 |
| *L. capsici* $P_{T5}$-*serp* | 608.58±238.17*** |

Results are shown as means ± standard deviations. The mean values were obtained in four independent experiments, each with two technical replicates. The two groups were compared using an unpaired two-tailed Student's *t*-test, $p = 2 \times 10^{-15}$.

**Supplementary Table S5.** Oligonucleotides used in this work for cloning and sequencing.

| Primers | Sequence | Purpose |
|---|---|---|
| **Oligonucleotides used for cloning** | | |
| T5_KpnI (for) | GGTACCGTGCCACCTGACGTCTAAG | To amplify a 1063 bp fragment containing T5 promoter – *gfp* – lambda t0 terminator from plasmid pTurboGFP-B |
| T5_XbaI (rev) | TCTAGACTGAAAATCTCGCCA AGCTAGC | |
| Gro_KpnI (for) | GGTACCCGGACCGACGCCTGTCA | To amplify a 199 bp promoter GroEL from *L. enzymogenes* VKM B-2235$^{T}$ DNA |
| Gro_HindIII (rev) | AAGCTTGGCGACCTCTGTAAGTAATTGAATTGG | |
| GFP_HindIII (for) | AAGCTTATGGAGAGCGACGAGAGCGG | To amplify a 705 bp *gfp* from plasmid pTurboGFP-B |
| GFP_BamHI (rev) | GGATCCTCATTCTTCACCGGCATCTGC | |
| Term_BamHI (for) | GGATCCCAAATAAAACGAAAGGCTCAGTCG | To amplify a 230 bp *rrnB* T1 and T2 transcriptional terminators from plasmid pEX18 |
| Term_XbaI (rev) | TCTAGAAGAGTTTGTAGAAACGCAAAAAGGC | |
| Gro_KpnI (for) | GGTACCCGGACCGACGCCTGTCA | To amplify a 200 bp promoter GroEL from plasmid PBBR1-MCS5 $P_{GroEL}$-*gfp* |
| Gro(A)_HindIII (rev) | AAGCTTTGGCGACCTCTGTAAGTAATTGAATTGG | |
| Blp1_HindIII (for) | AAGCTTATGAAGGCGATTTCGGGAGC | To amplify a 1143 bp *blp* from *L. capsici* VKM B-2533$^{T}$ DNA |
| BlpI_BamHI (rev) | GGATCCTCAGTTCGGGCCTGGG | |
| Blp2_BamHI (for) | GGATCCATGAAGGCGATTTCGGGAGC | To amplify a 1143 bp *blp* from plasmid PBBR1-MCS5 $P_{GroEL(A)}$-*blp* |
| Blp2_HindIII (rev) | AAGCTTTCAGTTCGGGCCTGGG | |
| Serp_BamHI (for) | GGATCCATGATCCGCAAGAACGCACTTTG | To amplify a 1383 bp *serp* from *L. capsici* VKM B-2533$^{T}$ DNA |
| Serp_HindIII (rev) | AAGCTTTCA GGGATTGAAATAGCTCGACACG | |
| **Oligonucleotides used for sequencing** | | |
| Gro_KpnI (for) | GGTACCCGGACCGACGCCTGTCA | To confirm the correctness of the cloned fragments and the absence of random mutations in the constructed vectors PBBR1-MCS5 $P_{GroEL}$-*gfp*, PBBR1-MCS5 $P_{GroEL(A)}$-*gfp*, PBBR1-MCS5 $P_{GroEL(A)}$-*blp* |
| Term_XbaI (rev) | TCTAGAAGAGTTTGTAGAAACGCAAAAAGGC | |
| T5_KpnI (for) | GGTACCGTGCCACCTGACGTCTAAG | To confirm the correctness of the cloned fragments and the absence of random mutations in the constructed vectors PBBR1-MCS5 $P_{T5}$-*gfp*, PBBR1-MCS5 $P_{T5}$-*blp*, PBBR1-MCS5 $P_{T5}$-*serp* |
| T5_XbaI (rev) | TCTAGACTGAAAATCTCGCCAAGCTAGC | |

**Supplementary Table S6.** Concentration of DNA template for PCR reaction.

| DNA template | Final concentration, ng/μL | Purpose |
|---|---|---|
| pTurboGFP-B | 1.58 | To amplify a 1063 bp fragment containing T5 promoter – *gfp* – lambda t0 terminator |
| *L. enzymogenes* VKM B-2235$^{T}$ DNA | 0.14 | To amplify a 199 bp promoter GroEL |
| pTurboGFP-B | 1.58 | To amplify a 705 bp *gfp* |
| pEX18 | 8.39 | To amplify a 230 bp *rrnB* T1 and T2 transcriptional terminators |
| PBBR1-MCS5 $P_{GroEL}$-*gfp* | 3.18 | To amplify a 200 bp promoter GroEL with modification |
| *L. capsici* VKM B-2533$^{T}$ DNA | 0.31 | To amplify a 1143 bp *blp* for construction of PBBR1-MCS5 $P_{GroEL(A)}$-*blp* |
| PBBR1-MCS5 $P_{GroEL}$-*blp* | 1.94 | To amplify a 1143 bp *blp* for construction of PBBR1-MCS5 $P_{T5}$-*blp* |
| *L. capsici* VKM B-2533$^{T}$ DNA | 0.31 | To amplify a 1383 bp *serp* |

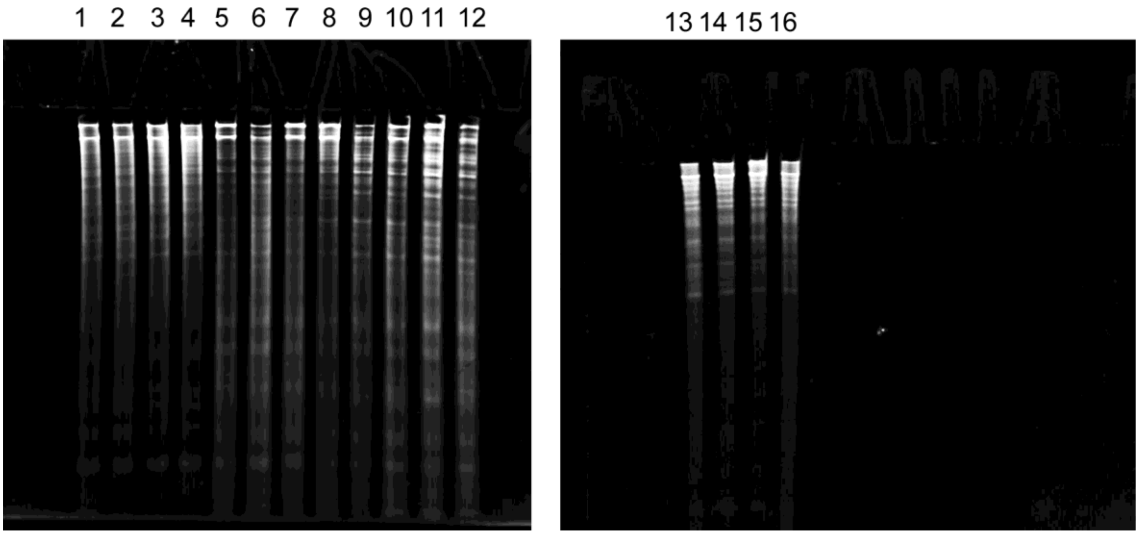


**Supplementary Figure S13.** 4% PAG with 8 M urea: 1, 2, 3, 4 – samples of wild-type *L. capsici* RNA; 5, 6, 7, 8 – samples of *L. capsici* $P_{GroEL(A)}$-*blp* RNA; 9, 10, 11, 12 – samples of *L. capsici* $P_{T5}$-*blp* RNA; 13, 14, 15, 16 – samples of *L. capsici* $P_{T5}$-*serp* RNA.

**Supplementary Table S7.** Oligonucleotides used in this work for RT-qPCR.

| Gene | locus tag (Protein id) | Sequence | Amplicon, bp | PCR efficiency |
|---|---|---|---|---|
| *gntR** | IEQ11_RS12995 (WP_036104172.1) | GntR_(for) TATCGCCAGCTCAAGGAACG GntR_(rev) CGCGAGACAGTGATGGGATT | 125 | 1.97 |
| *alpA* | IEQ11_RS15580 (WP_191823338.1) | AlpA_(for) CTGCAGACCGAAAAACTCGC AlpA_(rev) GCAACTGCTTGAGGCTATGC | 196 | 1.94 |
| *blp* | IEQ11_RS04180 (WP_191821694.1) | Blp_(for) GGGAGCAAGAATCACGCTGT Blp_(rev) GTGCTTGGCCAGATAGGTG | 154 | 1.92 |
| *serp* | IEQ11_RS08595 (WP_191822808.1) | Serp_(for) ATCCGCAAGAACGCACTTTG Serp_(rev) GGATCGAGAAGCAGGACAGG | 58 | 2.07 |

* reference gene
